# Development and Adaptation of Iranian Youth Reproductive Health Questionnaire

**DOI:** 10.1155/2013/950278

**Published:** 2013-07-31

**Authors:** Abbas Mousavi, Afsaneh Keramat, Katayon Vakilian, Safar Ali Esmaeili Vardanjani

**Affiliations:** ^1^Research Center of Psychiatry, Golestan University of Medical Scinces, Golestan, Iran; ^2^School of Nursing and Midwifery, Shahroud University of Medical Scinces, Shahroud, Iran; ^3^Candidate of Reproductive Health, School of Nursing Midwifery, Sharhoud University of Medical Sciences, Sharhroud, Iran; ^4^Nursing Education, Shahrekord University of Medical Sciences, Shahrekord, Iran

## Abstract

Iran is a young country, and sexual behavior is shaped in this period. This research aimed to provide an assessment tool to evaluate Iranian youth reproductive health. This multistage research was conducted to design a valid questionnaire in the domains of knowledge, attitude, and behavior of the youth in order to evaluate behavior change programs. For this reason, after conducting a careful literature review and a qualitative research, the questionnaire was prepared. Forward and backward translations were performed. Professionals and students were used to make sure of qualitative and quantitative content and face validity. After conducting the pilot study on 100 students and eliminating defects in performance, reliability was evaluated by test-retest and Cronbach's alpha was calculated. In this study, out of 268 questions, 198 were retained after face and content validity. Self-efficacy of communication with father and mother, self-efficacy of condom use, and self-efficacy of abstinence had the highest Cronbach's alpha. Moreover, communication with parents regarding reproductive health issues and attitude to abstinence had a high Cronbach's alpha, as well. It seems to be a good instrument for assessment of Iranian reproductive health, and we are going to assess youth reproductive health in the future.

## 1. Introduction

Iran is a relatively young country; according to the 2006 national census conducted by Statistical Centre of Iran, about 15.2% of the Iranian population (about 17,700,000 people) are in the age range 15–24 [1]. Young adults and adults comprise one of the high risk groups of every society [2]. Adolescence is the era of transition to adulthood and many individuals start sexual behaviors in adolescence which may result in negative outcomes such as teenage pregnancy, teenage parenthood, and sexually transmitted diseases like AIDS [3]. In 2000, about 20,000 young age pregnancies were reported in girls aged 10–14 which resulted in spontaneous abortion in 14%, live birth in 43%, and selective abortion in 43% [4]. 

On the other hand, AIDS is a health, social, and psychological crisis which has roots in high risk behaviors. It affects both adults and children as it could be stated that AIDS is now the problem of the young people of whom 85% live in developing countries [5, 6]. In Iran, also, the third wave of AIDS, which is sexual transmission, is increasing. According to a report from the Communicable Diseases Office of the Iranian Ministry of Health and Medical Education, the number of the patients with sexually transmitted diseases (except for AIDS) increased from 19044 in 1996 to 150429 in 2004. Moreover, the number of AIDS patients increased from 40 in 1991 to 23125 in July 2011 [7]. In the 21st special session of the International Conference on Population and Development (+5), it was decided that HIV infection rates in persons 15–24 years of age should be reduced by 25 percent in the most affected countries by 2005 and by 25 percent globally by 2010. Also, by 2005, 90% of young men and women should have access to information, education, and services necessary to develop the life skills required to reduce their vulnerability to HIV infection [8].

Research shows that reproductive health programs are conducted with a special focus on AIDS in schools, media, and the society formally and informally [9, 10], but the efficacy of such programs has not been largely evaluated. One of the reasons may be the lack of a comprehensive and efficient instrument proportional to the program objectives. 

This study was conducted to design a domestic instrument for the evaluation of the Iranian young people considering behavior change theories that include behavior-related variables.

## 2. Material and Methods 

At first, researchers designed a questionnaire development in 9 steps, described in the following. 

*Step  1.* The ethical committee of Shahroud Univerity of Medical Sciences approved the study. Then, a literature review was conducted to identify published surveys related to adolescent health with a special focus on sexual and reproductive health through search engines such as Google Scholar, Alta Vista, Google, and many other databases (PubMed, Science Direct, Proquest, CINAHL) available as electronic libraries. 

More than 20 MESH terms such as young people, youth, adolescent, adolescence, teenage, behavior, parent, mother, father, self-efficacy, knowledge, attitude, HIV/AIDS, sexual behavior, sexual relationship, STD, religious, parent relationship, sexuality, program, behavior surveillance system, reproductive health, instrument, and scale. were searched alone or in combination. Also, WHO, FHI, CDC, and UNAIDS websites were searched in order to access questionnaires developed for youth reproductive health. We made a catalogue of pertinent self- or interviewer-administered questionnaires used in previous surveys on young individuals, particularly in African settings. Three questionnaires developed by WHO, FHI, and CDC were used for this study because most researchers and behavior surveillance systems use these questionnaires for the evaluation of reproductive health programs in the youth. At this point, coverage of a vast range of issues, scales, and questions that would be modified after peer review received emphasis. Items that measured attitude toward delay of sexual intercourse and the use of condom, knowledge about HIV/AIDS, relationship with parents, beliefs about HIV/AIDS, health seeking behavior, self-efficacy, subjective norms, behavior, and exposure to HIV/AIDS information by media were included. Care was taken to include all items and scales pertinent to the objectives of the study. In the end, considering the aforementioned, we had questionnaire with 208 items covering a wide range of variables.

*Step  2.* Along with the literature review, a qualitative study was done in order to discover university students' opinions regarding premarital friendship factors and current high risk sexual behaviors in university students and youth people in the society, their attitudes and beliefs about premarital friendship, and community barriers to access reproductive health for young people. This qualitative research helped us to modify our questionnaire based on the subculture of the young people and important domains in reproductive health, expressed in focus groups. Sampling process, data collection and analysis of the 38 volunteers willing to participate in this present research were selected through a purpose-based approach. Before participation, all participants signed informed consent forms. The participants were 18–24 years of age (20 ± 1.2 for girls and 21 ± 1.5 for boys). After analysis, we noted that students pointed to domains such as knowledge of reproductive health in HIV/AIDS, contraception, sexual transmitted diseases, attitude (premarital friendship and sexual behaviors and parents' attitude towards reproductive health), skills (family life skills and negotiation with parents about reproductive health), behaviors (high risk behaviors, alcohol and drug use, and outcome of sexual behaviors in Iran), the roles of friends and institutions in sexual health (school, health services, health providers' skills), social taboos and barriers to reproductive health.

*Step  3.* Three questionnaires belonging to the Center for Disease Control, World Health Organization, and Family Health International were assessed by the research team in 3 sessions in order to select questions compatible with the Iranian culture.

**Figure 1:**
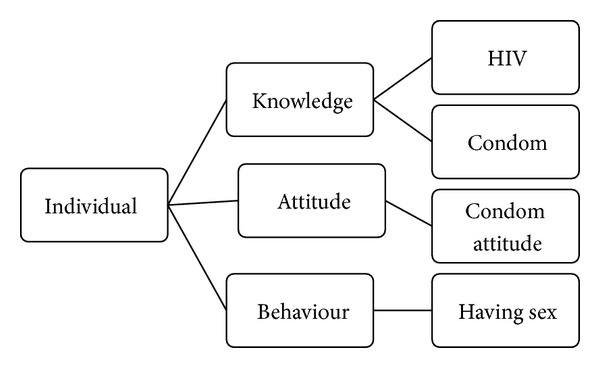
Category individual and its sub categories.

**Figure 2:**
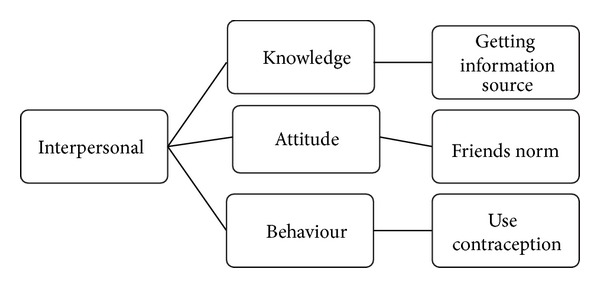
Category interpersonal and its sub categories.

**Figure 3:**
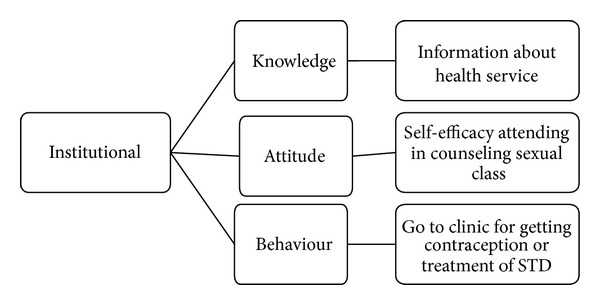
Category institutional and its sub categories.

*Step  4  (drawing map).* We drew the map in 3 main categories of individual, interpersonal, and institutional. Each main category included 3 subcategories of knowledge, attitude, and behavior. These were showed in Figures 1, 2, and 3.

*Step  5  (translation and back translation).* The resulting questionnaire with 268 items in English was translated into Persian. The translations were done by professionals who were familiar with medical and social terminology. To check for the accuracy of the translation and to ensure that the original concept was preserved, the translated versions were then translated back into English by individuals who had not been previously involved in the questionnaire development and translation process. The research team checked backward translations to ensure that each question, instruction, and response option was accurate. Items with apparent discrepancies between the two versions were then modified.

*Step  6  (content validity).* In order to identify and finalize criteria, prioritize issues, and define suitable items/scales for inclusion in the instrumentation, the instrument, study objectives, and a check list of content validity index Waltz and Bausell [11] emailed to 5 experts who were faculty members in different medical universities, such as Tehran University of Medical Sciences, Shahid Beheshti University of Medical Sciences, and Shahroud University of Medical Sciences, in fields like reproductive health, psychiatry, obstetrics, and midwifery. The professors were asked to highlight the most important and relevant domains related to reproductive health in Iranian young people. Also, to establish face and content validity, the questionnaire was given to 14 selected professors who were experienced and renowned in their fields. 

The questions and response options were checked for vocabulary, culture, language, and age appropriateness. We also made sure that the items were consistent with the constructs of the theoretical framework. For each scale and each domain, items and questions that fulfilled the criteria of relevance and appropriateness were retained.

*Step  7  (pretest with students).* To further assess face and content validity, the instrument was piloted with small samples of university students in different fields of study. The questionnaire was tested for readability, relevance, language, comprehension, and cultural and age appropriateness. Ten female and 10 male students completed the questionnaire individually. They were requested to take note of or ask about anything in the questionnaire that they had difficulty understanding. The researcher also took note of questionnaire items and instructions with which learners appeared to have difficulties so that they could be rechecked.

Group discussions were held immediately after completion of the questionnaire. The probing technique was used to detect the meaning of each question and to identify problematic items.

Based on this feedback, the instruments were revised, translated and back translated accordingly.

*Step  8  (pilot test).* The pilot test was done on 100 university students who were randomly selected. The purpose of the field test was to assess the feasibility and the time needed to answer the questions, arrange questions, and receive students' feedbacks. Every questionnaire took learners up to 45–60 minutes to complete. As a result of the piloting survey, a small number of changes were made to the questionnaire. The arrange and format of the questions were changed after receiving feedback and the final format was determined.

*Step  9  (reliability tests).* The reliability of the instrument was determined by test-retest to determine the level of agreement between responses after 10 days. The questionnaire was administered in one of the universities in Shahroud by one of the researchers. This questionnaire, consisting of 198 items, measured potential determinants of reproductive health and HIV knowledge, attitude, friends' values and norms, norms, self-efficacy, outcome expectancy, communication with parents, friends, and the opposite sex. To link data for each respondent at test-retest, a unique number was assigned to each questionnaire at the first administration

The students opened their envelopes and entered this number onto the new questionnaire.

Sexual behavior related questions, which were very sensitive questions, were not included in the reliability assessment of test-retest because in the next step of the research, internal consistency was measured by Cronbach's alpha correlation coefficient and included 500 randomly selected participants.

## 3. Result

 For content validity, culturally appropriate substitutes were used for words such as sex, withdrawal, sexually transmitted diseases, vaginal sex, penis, and vagina. Moreover, in response to the question “how do you feel when you discuss reproductive health issues with your parents, relatives or health professionals?”, the participants were asked to use a 5-point Likert scale (from absolutely not comfortable to absolutely comfortable) instead of very good, good, okay, bad, and very bad. 

For content validity, domains and questions for content validity rate (CVR) less than 0.6 were deleted. For example, the domain of adolescents' attitude toward discussing reproductive health issues with parents included 10 questions. 

All together, 41 questions were left out, all after conferring with team members and therefore 227 questions along with domains with high CVR were retained.  Table 1 shows some of the domains.

After CVR, the questionnaire was returned to the professors for content validity index (CVI) assessment and they were asked to comment on the pertinence and clarity of each of the 227 items using a 4-point Likert scale and also give their opinion on their cultural appropriateness in writing. Only questions with CVI more than 0.71 entered the study [12]. In the domain of reproductive health knowledge, the professors believed that 4 questions about STDs and their diagnosis and treatment were assumed vague for students. However, these questions were not deleted but face validity, pretest and pilot study showed that students had no information in those areas and the questions were unanswered. Therefore, these 4 questions were deleted but, instead, a question on the symptoms of STD was added. 

Questions with CVI less than 0.6 for clarity were rephrased to become understandable for the students, and 23 questions with CVI less than 0.71 were deleted. The reviewers believed that the questionnaire was too long and suggested that questions that addressed details of the sexual relationship or its reasons or were culturally inappropriate be deleted In this regard, after conferring with advising professors and conveying the comments of the reviewers, 29 questions were deleted to decrease the number of the questions to 198. 

On students' pretest, other questions which were differently understood by students and the researcher were modified for a similar understanding. 

After pretest, to assess feasibility, a pilot study was conducted on 100 individuals. This pilot showed that the response rate increased with the presence of the researcher to provide full explanation on the research objective and offering a reward for completing the questionnaire. After analysis and obtaining feedback from students, the format of the questionnaire and questions order were modified and easier less sensitive questions were placed first. 

Findings of reliability shown in Table 2.

## 4. Discussion

As discussed earlier, questions were derived from WHO, World Bank, UNAIDS, and FHI questionnaires and review of the literature. 

In this instrument, questions about reproductive health, including questions on pregnancy physiology, AIDS/STD, contraception, and condom, were obtained from the WHO questionnaire. On the physiology of pregnancy, 1 question was deleted and 3 were included in the questionnaire. Regarding the knowledge of HIV/AIDS, all 4 questions entered the study. Four questions in the WHO questionnaire addressed STDs; we modified the question “do you have information about sexually transmitted diseases?” by adding the name of 9 diseases like gonorrhea, syphilis, and so forth. A positive answer to each disease, indicating having information on that disease, was assigned 1 score (a total of 9 scores if the participant had information on all 9 diseases). Moreover, 1 score was given to the knowledge of every symptom of sexually transmitted diseases. 

Regarding contraception knowledge, all 13 questions of the WHO questionnaire were included in our questionnaire. 

Knowledge of condom had 6 questions; the question “condom disappears inside a woman's body” was deleted in content validity and the remaining 5 questions were added to the questionnaire. 

Questions that dealt with knowledge had three choices (yes, no, I have no idea); a correct answer was scored 1 and a wrong answer or “I have no idea” was scored zero.

The CDC questionnaire was employed for knowledge of AIDS. It contained 15 questions but only 14 were included in our questionnaire since the 15th question of the CDC questionnaire was deleted. A 5-point Likert scale was used for scoring (from I am completely sure it is correct to I am completely sure it is incorrect) with a total score of 90. According to the obtained total score, knowledge of AIDS was categorized as good, intermediate, and little.

Regarding attitude toward HIV and evaluation of the stigma attached to HIV positivity in the society, the CDC questionnaire with 8 questions was employed and participants used a 5-point Likert scale to answer the questions with a total score of 40 and according to the received score, and the attitude was categorized as positive, negative, and neutral. Attention was paid to some questions that required reverse scoring.

The CDC questionnaire was also used to assess adolescents' perceived threat of AIDS. There were 5 questions in this domain which were answered using a 5-point Likert scale with a total score of 25. Based on the received score, the perceived threat was categorized as weak, moderate, and good. 

To answer the questions regarding the attitude towards appropriate age for marriage adopted from the FHI questionnaire, the participants were asked to choose an answer indicating a specific age rather than age range. 

Regarding the attitude toward child bearing immediately after marriage, one question was added to the questionnaire and participants used a 5-point Likert scale (from I totally agree to I totally disagree) to indicate their answers.

Regarding the attitude toward the number of children, the question “what is appropriate number of children to have” was used.

The attitude toward sex before marriage was assessed using the WHO questionnaire with 9 questions, which were also previously used by Mohammadi et al. [13]. One question stating “sexual contact with the same gender is inappropriate” was deleted in content validation and therefore 8 questions in a 5-point Likert scale and a total score of 40 were included in the study. According to the obtained score, the attitude was categorized as weak, intermediate, and good. 

Regarding the attitude toward condom, the CDC questionnaire with 5 questions in a 5-point Likert scale and a maximum score of 25 was employed and accordingly the attitude was categorized as weak, intermediate, and good. 

Regarding self-efficiency of condom use and abstinence, a questionnaire developed by Kaljee et al. [14] was used for its better cultural congruity after obtaining authorization from the author. There were 5 questions about condom use self-efficiency and 8 questions about abstinence self-efficiency, both designed in a 5-point Likert scale, with total scores of 25 and 40, respectively. According to obtained score, self-efficiency was categorized as weak, intermediate and good. 

Regarding the friends' norms, the CDC questionnaire with 7 questions was employed; moreover, considering the focus groups in the qualitative study, 2 more questions, “how many of your friends do you think have girlfriends/boyfriends?” and “how many of your friend have anal sex?”, were added to the questionnaire. A 4-point Likert scale including “all,” “most,” “some” and “none,” was used to answer the 9 questions. Each question was analyzed individually. 

Regarding the perception of friends' values, the CDC questionnaire with 3 questions was used. One question, “in your opinion, how many of your friends believe that sex with someone of the same gender is inappropriate?” was added to the list of the questions based on the qualitative study. The questions were answered in a 4-point Likert scale including “all,” “most,” “some,” and “none.” Each of the 4 questions was analyzed individually.

Regarding talking about reproductive health issues with friends, parents, health providers and friends of the opposite sex, 4 questions of the FHI questionnaire were used and participants were asked to answer the questions with “yes,” “no,” or “I do not remember.” The same questions were also used to evaluate comfort with parents and health providers and a 5-point Liker scale (from I am totally comfortable to I am totally uncomfortable) with a total score of 25 was used to answer the questions. Based on the obtained score, 3 categories of “good,” “intermediate,” and “weak” were recognized. 

The rest of the questions, which addressed demographic data and variables affecting reproductive health, were also present in other youth reproductive health questionnaires. 

Religious questions comprised “visiting religious places” and “saying prayer” to assess public and private aspects of religiosity, respectively. Religious salience was evaluated with the question “how does religion affect sexual behaviors?” These questions have also been used in many studies and are available in the FHI questionnaire.

The results showed that the correlation coefficient and Cronbach's alpha of the comprehensive knowledge of HIV were *r* = 0.72 and *α* = 0.60, respectively. In a study that was conducted on Spanish students to evaluate the reliability of the same questionnaire, the correlation coefficient and Cronbach's alpha were found to be 0.59 and 0.57, respectively. The researcher attributed the lower correlation coefficient to the fact that each question had 2 correct answers; although these types of questions prevent responders from guessing the correct answer, they decrease the variance of the scale. Moreover, the lower Cronbach's alpha was attributed to the correct answers of 90% of the participants and the resultant lower variance and alpha [15]. Furthermore, the correlation coefficient and Cronbach's alpha of perceived threat of AIDS, peer pressure, and attitude to condom were 0.49 and 0.30, 0.62 and 0.55, and 0.17 and 0.0, respectively, in the same study [15] which was not congruent with other studies [16, 17]. 

In the present study, the correlation coefficient and Cronbach's alpha were 0.68 and 0.61 from friends' norm, 0.72 and 0.61 for attitude to condom and 0.94 and 0.62 for perceived threat of AIDS, respectively, which were higher than those reported by Zometa [15]. Only the attitude to condom had an acceptable test-retest reliability.

Regarding the WHO questionnaire, although Mohammadi et al. [9] used some parts of it in Iran, they did not determine its reliability while the present study evaluated its reliability and showed a correlation coefficient of 0.62 for reproductive health knowledge. In the subgroups of reproductive health knowledge, the Cronbach's alpha was 0.69 for reproduction physiology, 0.65 for contraception, −0.6 for HIV/STD, 0.6 for contraception, and 0.60 for condom knowledge, indicating intermediate reliability. 

Skevington believes that indigenizing always affects reliability because the properties of a source instrument are compared with those of a new translation in a target population and the parameters of reliability and validity tend to be poorer in the target population when compared to the source. In successful indigenization, before implementing the items in the target population, they are tested in the new population and necessary revisions are made [18].

The reliability results of the self-efficiency of condom use and abstinence showed a high Cronbach's alpha. This method was also used in a study conducted by Kaljee et al. [14] but the Cronbach's alpha was not reflected in their report.

## 5. Conclusion

Our questionnaire was found to be valid. However, the reliability of the questionnaire was moderate in some domains.

This questionnaire is a comprehensive instrument and the researchers and policy makers can employ the items and domains of this questionnaire in their researches and programs. Since this questionnaire is adopted from internationally recognized instruments, it can be used for the evaluation of reproductive health in schools, youth friendly clinics, and related national surveys in the youth.

## Figures and Tables

**Table 1:** Domain assessment of youth reproductive health by content validity rate.

Domain		CVR
Individual knowledge, attitude, and self-efficacy	Reproductive health knowledge	0.60
	Comprehensive HIV knowledge	1
	Attitude to people infected by HIV	0.60
	Perception susceptibility of young people to HIV	0.60
	Attitude towards marriage age	1
	Premarital sex attitude	1
	Condom attitude	0.60
	Attitude towards child bearing immediately after marriage	0.60
	Condom self-efficacy	1
	Abstinence self-efficacy	0.60

Behavior and outcome	Having sex	0.60
	Having pregnancy	0.60

Family	Attitude to talking to parents about reproductive health issue	0.80
	Talking to mother about reproductive health	0.80
	Talking to father about reproductive health	0.80
	Self-efficacy talking to mother and father about reproductive health	0.80
	Talking to a friend about reproductive health	0.60
	Talking with opposite sex friend about reproductive health	0.60
	Friends norm	0.60

Health provider institutional	Self-efficacy for sex consult	0.60
	Self-efficacy to visit to STD	1
	Talking to health provider about reproductive health issues	0.60
	Go to clinic for reproductive health service	0.60
	Exposure to the reproductive health message through national mass media	0.60
	Exposure to pornography	0.60
	Exposure to violent media	0.60

**Table 2:** Reliability tests of youth reproductive health questionnaire.

Domains	Test—*R*-test	Cronbach's alpha
Reproductive health knowledge		
Physiology of pregnancy and contraception	*r* = 0.62	0.69
Contraception		0.65
HIV/STD		0.60
Condom knowledge		0.60
Comprehensive HIV knowledge	*r* = 0.72	0.60
People infected by HIV attitude	*r* = 0.65	0.60
Perception susceptibility young people to HIV	*r* = 0.94	0.60
Abstinence attitude	*r* = 0.60	0.87
Condom attitude	*r* = 0.72	0.61
Abstinence self-efficacy	*r* = 0.65	0.81
Condom self-efficacy	*r* = 0.67	0.80
Friends norm	*r* = 0.68	0.61
Talking to friend about reproductive health		0.62
Talking to mother about reproductive health		0.77
Talking to father about reproductive health		0.82
Self-efficacy talking to mother about reproductive health		0.94
Self-efficacy talking to health provider about reproductive health issues		0.75
Self-efficacy talking to father about reproductive health		0.93
STD sign		0.65
